# Surgical Instruments

**Published:** 1855-01

**Authors:** 


### SURGICAL INSTRUMENTS.

We would call the attention of our readers to the advertisement of K. Klott, of Columbus, Ohio. Mr. Klott forwarded us a specimen of his instruments by express, and we are fully prepared to sustain the very favorable impression made in his favor by a letter from a friend in that city. The specimen before us is a small but choice case for the every day practitioner, a desideratum much needed, as the ordinary sized case is too large and too heavy for convenience. This waives that objection, and yet there are contained in it all the instruments usually required. The form of the instruments, their superior finish, and the quality of the metal, particularly speak for Mr. Klott as a very superior workman.

